# Stacking-ac4C: an ensemble model using mixed features for identifying n4-acetylcytidine in mRNA

**DOI:** 10.3389/fimmu.2023.1267755

**Published:** 2023-11-29

**Authors:** Li-Liang Lou, Wang-Ren Qiu, Zi Liu, Zhao-Chun Xu, Xuan Xiao, Shun-Fa Huang

**Affiliations:** ^1^ Computer Department, Jing-De-Zhen Ceramic Institute, Jingdezhen, China; ^2^ School of Information Engineering , Jingdezhen University, Jingdezhen, China

**Keywords:** N4-acetylcytidine, feature extraction, stacking heterogeneous integration, Cluster Centroids algorithm, ensemble model

## Abstract

N4-acetylcytidine (ac4C) is a modification of cytidine at the nitrogen-4 position, playing a significant role in the translation process of mRNA. However, the precise mechanism and details of how ac4C modifies translated mRNA remain unclear. Since identifying ac4C sites using conventional experimental methods is both labor-intensive and time-consuming, there is an urgent need for a method that can promptly recognize ac4C sites. In this paper, we propose a comprehensive ensemble learning model, the Stacking-based heterogeneous integrated ac4C model, engineered explicitly to identify ac4C sites. This innovative model integrates three distinct feature extraction methodologies: Kmer, electron-ion interaction pseudo-potential values (PseEIIP), and pseudo-K-tuple nucleotide composition (PseKNC). The model also incorporates the robust Cluster Centroids algorithm to enhance its performance in dealing with imbalanced data and alleviate underfitting issues. Our independent testing experiments indicate that our proposed model improves the Mcc by 15.61% and the ROC by 5.97% compared to existing models. To test our model’s adaptability, we also utilized a balanced dataset assembled by the authors of iRNA-ac4C. Our model showed an increase in Sn of 4.1%, an increase in Acc of nearly 1%, and ROC improvement of 0.35% on this balanced dataset. The code for our model is freely accessible at https://github.com/louliliang/ST-ac4C.git, allowing users to quickly build their model without dealing with complicated mathematical equations.

## 1 Introduction

To date, over 170 modified nucleosides have been found in RNA. These post-transcriptional modifications play a significant role in molecular interactions and intermolecular relations. Introducing subtle structural changes contributes to RNA’s functional diversity by regulating translation efficiency, mRNA stability, and RNA-protein interactions – all factors that are vital for cellular growth and development (1, 2). Ac4C has been linked with various human diseases, including inflammation, metabolic disorders, autoimmune diseases, and cancer (3). Identifying and examining ac4C sites are critical areas in biological and bioinformatics research. In early studies, the identification of ac4C sites was mainly done through experiments such as high-performance liquid chromatography (HPLC) and HPLC-mass Spectrometry. However, as these experimental methods require substantial time to detect ac4C in mRNA, there is an urgent need for computer-based methods that can identify ac4C sites accurately and reliably.

**Figure 1:**
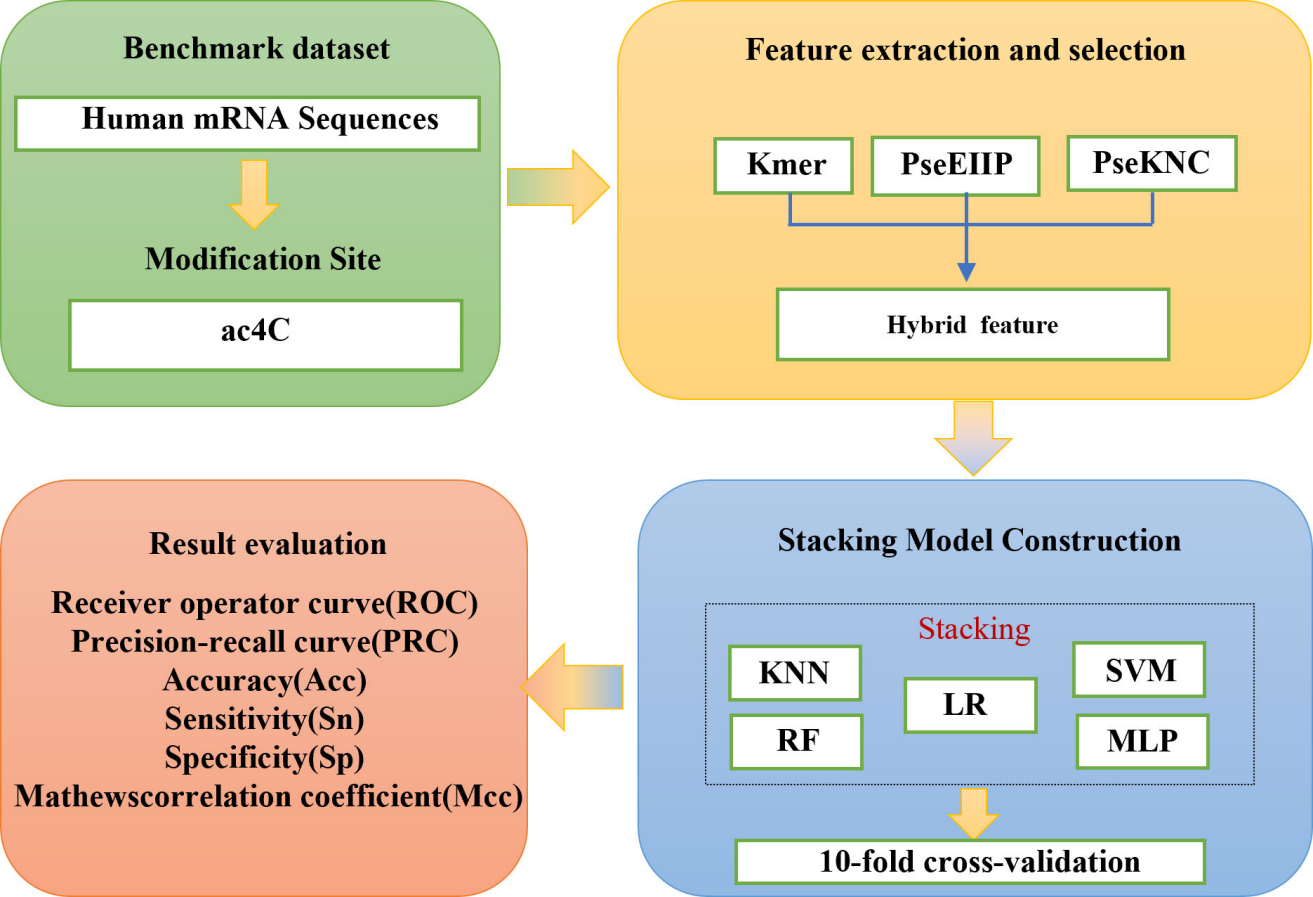
The scheme diagram for establishing Stacking-ac4C.

In recent years, four computational methods have been developed to identify ac4C sites in human mRNA. The first one, PACES, was developed by Zhao et al. (4). PACES utilizes position-specific dinucleotide sequence Spectra and K-nucleotide frequencies as coding methods, with random forest (RF) (5) deployed as a training model to yield the outcomes. PACES (4) achieved an area under the characteristic curve and the exact recall curve of 0.874 and 0.485, respectively. The second approach is an integrated model (XGBoost) (6, 7) proposed by Alam for predicting ac4C locations. The ROC and PRC of the XGBoost model are 0.889 and 0.581, respectively. Subsequently, the third method is Wang’s DeepAc4C (8) model, which is built based on a convolutional neural network (CNN) (9) and a hybrid feature that integrates physicochemical patterns and nucleic acid distribution. The last prediction method is a gradient boosting decision tree (GBDT) (10, 11) based on Kmer (12) nucleotide composition, nucleotide chemistry NCP (13), cumulative nucleotide frequency ANF (14), and minimum redundancy maximum correlation mRMR (15). The model achieved ROC values of 0.875 and 0.880 on the training and independent test datasets, respectively. Despite these models showing commendable performance, considerable scope remains for enhancing the predictive efficacy of all models, as mentioned earlier. To improve the performance of ac4C site identification, we have proposed a novel method based on integrated learning Stacking (16) called Stacking-ac4C, as shown in **Figure 1**. This approach consolidates K nucleotide composition Kmer, electronic energy PseEIIP (17) based on normalized trinucleotide frequencies and four nucleotides, along with trinucleotide occurrence frequencies and six physicochemical indicators PseKNC (18). The ROC of the proposed model on the cross-validation and independent test datasets were 0.9540 and 0.9487, respectively, which shows excellent performance compared to all the predictors, as mentioned earlier.

## 2 Materials and methods

### 2.1 Data collection and preprocessing

To develop a valuable and unbiased model, we extracted the data from PACES (4), available at http://www.rnanut.net/paces/. These data were also used for training and testing the models of XG-ac4C (7) and DeepAc4C (8), initially extracted by Danial Arango from 2134 genes with positive and negative ac4C sites. All of these genes were experimentally validated by high-throughput acRIP seq. This study’s training dataset consists of 1160 positive and 10855 negative samples. The independent testing dataset comprises 469 positive samples and 4343 negative samples. To demonstrate the model’s portability, the results were validated using a dataset constructed from the iRNA-ac4C article, which was collected by Arango et al. (19). Data can be obtained from the website http://lin-group.cn/server/iRNA-ac4C/. During the experiment, the CD-HIT (20) tool was used to remove sequence pair similarity larger than 0.8. The training dataset we obtained consists of 2206 positive samples and 2206 negative samples. The independent testing dataset consists of 552 positive samples and 552 negative samples, which are balanced data. Finally, a balanced dataset with a sequence length of 201bp was obtained. The specific information about the dataset is shown in **Table 1**.

**Table 1:** Data source distribution table.

Data source	subdataset	Number of Positive Samples	Number of Negative Samples
PACES (4)	Training	1160	10855
	Testing	469	4343
	Total	1629	15198
iRNA-ac4C (10, 11)	Training	2206	2206
	Testing	552	552
	Total	2758	2758

### 2.2 Sample formulation

Once the benchmark dataset has been prepared for the study, the next important step is formulating the samples and extracting the best feature set for constructing a robust and superior computational model. In recent years, various feature encoding strategies have been used to form biological sequence fragments, such as PseKNC (17), One-hot (21, 22), physicochemical features, and word2vec (23–25). This study selected some of the most common feature encoding approaches, including six physicochemical feature encoding strategies and the frequency of occurrence of k-nearest neighbor nucleic acids, to describe RNA fragments. Below, we elaborate on their respective principles in detail.

#### 2.2.1 Kmer nucleotide composition

The main idea of Kmer is the frequency of *k* nucleotides in an RNA sequence. The RNA sequence R can be transformed into a vector with 4k dimensions by using the Kmer frequency as follows:


(1) $${\text{R}}_{\text{k}-\text{mer}}={\left[f_{1}^{k-mer},f_{2}^{k-mer},\ldots ,f_{i}^{k-mer},\ldots ,f_{4}^{k-mer}\right]}^{T}\;$$

Where $f_{i}^{k-mer}$ is the normalized frequency of occurrence of the ith Kmer nucleotide in the sample sequence, and T denotes the transposition of the matrix. $f_{i}^{k-mer}$ can be expressed as:


(2) $$f_{i}^{k-mer}=\frac{N\left(t\right)}{L-K-1}\;$$

where N(t) is the number of Kmer type t in the RNA sequence R.

#### 2.2.2 PseKNC

The pseudo-k-tuple composition PseKNC is similar to SCPseDNC (26) and SCPseTNC (27), while PseKNC contains the frequency of trinucleotide occurrences and fusion information of six physicochemical indicators. The PseKNC contains a k-tuple nucleotide composition, which can be defined as:


(3) $${\text{D}=[d}_{1}{,d}_{2},\ldots d_{4^{k}}{,d}_{4^{k+1}},\cdots {,d}_{4^{k+\lambda }}{]}^{T}$$

(4) $$\{\begin{array}{c} \frac{\omega \theta_{\mu -4^{k}}}{\sum_{i=1}^{4^{k}}f_{i}+\omega \sum_{j=1}^{\lambda }\theta_{j}},\left(4^{k}\leq \mu \leq 4^{k}+\lambda \right)\\ \frac{f_{\mu }}{\sum_{i=1}^{4^{k}}f_{i}+\omega \sum_{j=1}^{\lambda }\theta_{j}},\left(1\leq \mu \leq 4\right) \end{array}$$

where λ is the number of total count levels (or hierarchies) of correlations along the nucleotide sequence; $f_{\mu }\;\left(u=1,2,\ldots ,4k\right)$ is the frequency of nucleotides $\sum_{i=1}^{4^{k}}f_{i}=1$ , w is the factor that θ_j_ defined as:


(5) $$\theta_{j}=\frac{1}{L-j-1}\sum_{i=1}^{L-j-1}\theta (R_{i}R_{i+1},R_{i+j}R_{i+j+1}),(j=1,2,\ldots \lambda \;;\;\lambda <L)\;$$

Where $\theta \left(R_{i}R_{i+1}s,\;R_{i+j}R_{i+j+1}\right)$ can be defined as:


(6) $$\theta \left(R_{i}R_{i+1},R_{i+j}R_{i+j+1}\right)=\frac{1}{\mu }\sum_{v=1}^{\mu }{\left[P_{v}\left(R_{i}R_{i+1}\right)-P_{v}\left(R_{i+j}R_{i+j+1}\right)\right]}^{2}$$

μ is the number of physicochemical indices, i.e., six indices (“rise”, “roll”, “translate”, “slide”, “slide “tilt”, “twist”) are set as RNA sequences, and $P_{v}\left(R_{i}R_{i+1}\right)$ is the value of the corresponding physicochemical index ( v=1,2,…,μ ). The physicochemical index of the nucleotid $\left(R_{i}R_{i+1}\right)$ is at position *i*. $P_{v}\left(R_{j+j}R_{i+j+1}\right)$ indicates the corresponding value of the nucleotide $\left(R_{j+j\;}R_{i+j+1}\right)$ at position *i* + *j*.

#### 2.2.3 PseEIIP

The electron-ion interaction pseudo-potential (EIIP) values for nucleotides A, G, C, and T are as follows: A (0.1260), C (0.1340), G (0.806), and T (0.1335) (28). The EIIP values for the nucleotides A, T, G, and C are denoted as EIIPA, EIIPT, EIIPG, and EIIPC, respectively. The average EIIP values of the three nucleotides in each sample were used to construct the eigenvectors, and the formula can be expressed as:


(7) $$\text{V}=\left[EIIP_{AAA}\cdot f_{AAA},EIIP_{AAC}\cdot f_{AAC},\cdots ,EIIP_{TTT}\cdot f_{TTT}\right]\;$$

where f_xyz_ denotes the normalized frequency of the *i*-th trinucleotide, and $EIIP_{xyz}=EIIP_{x}+EIIP_{y}+EIIP_{Z}$ denotes the EIIP value of a trinucleotide and X,Y,Z∈[A,C,G,T] .The dimensionality of the vector representation is 64.

### 2.3 Feature fusion

Three feature codes, Kmer (12), PseEIIP (17), and PseKNC (17), were combined to describe the ac4C locus samples, 48-D, 84-D, and 64-D feature vectors were obtained, respectively. These feature vectors describe the adjacent positional correlation information of the sequence and enhance the extraction of sequence information by utilizing the physical and chemical properties of nucleotides. The hybrid features are obtained by fusing these features to reach 196-D. To investigate what feature fusion would arrive at the optimal training results, we compare the four combinations of PseKNC, Kmer, PseKNC+PseEIIP, and Kmer+PseKNC+ PseEIIPPSEEIIP encoded in the Stacking model validated by 10-fold cross-validation and measured by Sn, Sp, Acc, Mcc, ROC, and PRC, as shown in **Table 2**. Where the evaluation metrics (Acc, Mcc, ROC, PRC) derived from training the Stacking algorithm model with the coding approach (Kmer+PseKNC+PSEEIIP) in cross-validation are higher than the mean values of the evaluation metrics derived from training with the previous four coding approaches by 5.135%, 3.865%, 3.62%, and 4.575%. This may be because multiple feature sets can utilize the advantage of one of the local features to compensate for the disadvantage of another local feature due to compensating for the disadvantage of the other local feature, so that the individual local features can be fused more effectively, thus, significantly enhancing the robustness of multiple feature sets.

**Table 2:** Comparison of cross-validation performance between Stacking algorithms trained with different feature combinations.

Feature	Sn	Sp	Acc	Mcc	ROC	PRC
PseKNC	0.8455	0.7944	0.8188	0.6395	0.9071	0.8985
Kmer	0.8783	0.8280	0.8532	0.7062	0.9258	0.9112
PseEIIP	0.8616	0.8220	0.8410	0.6826	0.9220	0.9143
PseKNC+PseEIIP	0.8642	0.8049	0.8340	0.6692	0.9167	0.9066
Kmer+PseKNC+PseEIIP	**0.8922**	**0.8840**	**0.8881**	**0.7749**	**0.9541**	**0.9534**

### 2.4 Stacking classification algorithm

**Figure 2:**
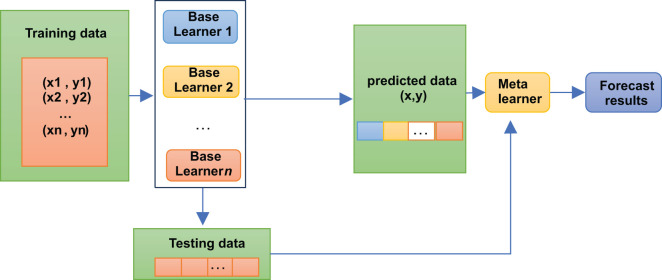
Stacking algorithm logic structure diagram.

A stacking model is not, strictly speaking, an algorithm but a strategy for model integration. As shown in **Figure 2**, the Stacking integration algorithm can be understood as a two-layer integration, where the first layer contains several base classifiers, also called base classifiers, which provide the predicted results (meta-features) to the second layer. In contrast, the second layer classifier is a logistic regression, which takes the results of the first layer classifiers as features to fit the predicted results.

The base classifiers of Stacking are usually obtained by training different learning algorithms. Stacking can also be considered a particular and specific combination strategy, typical of learning methods (16). In the Stacking training phase, the data used to train one layer of classifiers are also used to generate data for the second layer of classifiers, which runs the risk of overfitting. Thus, we used cross-validation to train the data and reduce this risk.

### 2.5 Imbalance data processing

The dataset of the PACES article is an unbalanced dataset with a ratio of positive and negative samples of 1 to 10, which results in the amount of information from the positive samples not being able to counteract the amount of information from the negative samples during the training of the Stacking-ac4C model, leading to a large number of misclassifications of the positive samples when the model is doing independent testing. Therefore, an algorithm is needed to reduce the imbalance between the number of positive and negative samples. Furthermore, data resampling is the most representative method to classify unbalanced data. In this study, we experimented with the two resampling widely adopted techniques: oversampling and undersampling (29). Due to the large number of negative samples of ac4C data with 415 nucleotide sequences in length, the use of the oversampling method is likely to lead to overfitting of the model, so we chose the cluster center method among the undersampling methods. This method first clusters the majority class samples using the K-means clustering algorithm and then reduces the number of majority class samples using the center of mass of each cluster to represent the clusters. **Table 3** compares the number of positive and negative samples before and after processing by the clustering centroid algorithm, and it can be clearly seen that after processing using the clustering centroid algorithm, the number of negative samples and the number of positive samples in the training set and test set are reduced to the same level, which indicates that the clustering centroid algorithm significantly reduces the number of negative samples of ac4C nucleotides.

**Table 3:** PACES dataset before and after imbalance treatment of the number of positive and negative samples in the dataset.

Whether or not imbalance treatment is performed	subdataset	Number of Positive Samples	Number of Negative Samples
no	Training	1160	10855
	Testing	469	4343
	Total	1629	15198
yes	Training	1148	1148
	Testing	467	466
	Total	1615	1614

### 2.6 Measures to prevent overfitting

To prevent the overfitting problem of the model, we used two measures. First, we used Stacking integrated learning, which is a method that combines the prediction results of multiple heterogeneous models to greatly reduce the variance of the model and avoid overfitting. Second, we added L2 regularization to the second layer of the LR model of the Stacking-ac4C model. Through L2 regularization, a “regular term” is added after the loss function to prevent overfitting of the model. This effectively prevents the model from assigning too much weight to any feature, thus helping to avoid overfitting (30).

### 2.7 Metrics formulation

To fully evaluate the performance of the model, 10-fold cross-validation and independent tests were used to evaluate the performance of the proposed model. In addition, the six metrics for evaluating the performance are specificity (Sp), sensitivity (Sn) (31, 32), Accuracy (ACC) (31, 32), correlation coefficient (MCC), the area under the receiver operating characteristic curve (ROC) (33, 34), and Precision-Recall Curve (PRC) (35–37), defined as follows:


(8) $$\{\begin{array}{l} Sn=\frac{TP}{TP+FN}\\ Sp=\frac{TN}{TN+FP}\\ Acc=\frac{TP+FN}{TP+FN+FP+TN}\\ Mcc=\frac{TP*TN-FP*FN}{\sqrt{\left(TP+FN\right)*\left(TN+FN\right)*\left(TP+FP\right)*\left(TN+FP\right)}} \end{array}\;$$

TP, FN, TN, and FP denote true positive, false positive, true negative, and false negative, respectively. Sn and Sp denote model correctness, Acc is used to measure the Accuracy between ac4C and non-ac4C sequences; Mcc is a metric commonly used to evaluate the classification performance of unbalanced data. In addition, since the data is imbalanced with a ratio of 1:10, another visual way to compare the current models is to compare the working characteristic ROC curves. The area under the ROC curve is also an important metric for assessing model performance. The higher the ROC, the better the performance.

## 3 Results

### 3.1 Classifiers combination

Models such as logistic regression (38), random forest (39), KNN (40), SVM (41), and neural networks have been experimented with and illustrated in paper XG-ac4C, paper iRNA-ac4C, and paper DeepAc4C; however, the results obtained using these models alone are not satisfactory; therefore, it is necessary to combine these models using a stacking approach. In Stacking Integration, selecting the optimal combination of base classifiers is an effective integrative learning strategy to improve the accuracy and robustness of the models. In this study, we used five standard machine learning algorithms as base classifiers, including logistic regression (Logistic), support vector machine (SVM), random forest (RF), k-nearest neighbor (KNN), and multilayer perceptron (MLP) (42) algorithms. To elucidate the learning advantage of the present model, we first evaluate the prediction performance of the base model on human AC4C locus data measured with 10-fold cross-validation and metrics mentioned in 3.5. Their optimal parameters are determined by a Bayesian net parameter learning method during the 10-fold cross-validation. This process can be implemented in Python using BaysSearchCV (43, 44), which tries all combinations of parameter values provided by the user and selects the best values from them. We attempted various parameter settings using the BaysSearchCV method and ultimately determined the optimal parameters. The optimal parameters for these classifiers are explained in **Table 4**. Subsequently, we selected five different single classifiers as the base classifier and then generated six combinations of the base classifiers.

**Table 4:** The optimal parameter settings for the Stacking-ac4c base model.

Base model	Best setting
LR	random_state=30, max_iter=1100
SVM	C= 1.6134, kernel=‘rbf’, degree=0.2651,tol=0.078
KNN	n_neighbors=20, leaf_size=17
RF	max_depth=10, min_samples_Split=10, min_samples_leaf=1, random_state=30
MLP	activation=‘relu’, alpha=1e-05, batch_size=37, beta_1 = 0.9, beta_2 = 0.999, epsilon=1e-08, hidden_layer_sizes=(11), learning_rate_init=0.021, max_iter=8532, momentum=0.58

We built an integrated model stacking to integrate all base models to get better results. The base and combined models are evaluated using independent test data. As shown in **Table 5**, the average Acc (0.8574) of the Stacking Integration Classifier model is improved by 7.22% compared to the average Acc (0.7852) of the Single Classifier model, which indicates that the Stacking Classifier, has better accuracy than the single classifier. The Stacking Integration Classifier model achieves an average Mcc and an average ROC of (0.71625) and (0.9289), increased by 13.18% and 5.87% more than the Single Classifier model, respectively, which indicates that the Stacking Integration Classifier model is more suitable for handling unbalanced data. The Stacking integrated model obtained better performance than the Single models, indicating that the integration model strategy improved the performance of the models, which may be because the Stacking Integration Classifier model uses different types of models for training, thus fusing the strengths of different models and improving the model’s generalization ability.

**Table 5:** Independent test performance comparison between different combinations of base classifiers on unbalanced datasets.

Classification model	Base-Classifier Combination	Sn	Sp	Acc	Mcc	ROC	PRC
Single classifier	(1) LR	0.8148	0.8051	0.8148	0.6297	0.8652	0.8374
	(2) Knn	0.4347	**0.9315**	0.6831	0.4219	0.8419	0.8115
	(3) SVM	0.8244	0.7473	0.7859	0.5734	0.8438	0.8092
	(4) RF	0.8672	0.8544	0.8608	0.7217	0.9295	0.9307
	(5) MLP	0.6788	0.8844	0.7816	0.5755	0.8706	0.8532
Stacking Integration Classifier	(1)+(2)+(3)+(4)	**0.8505**	0.8737	0.8621	0.7303	0.9303	0.9368
	(1)+(2)+(3)+(5)	0.7388	0.8694	0.8041	0.6134	0.8766	0.8514
	(1)+(2)+(4)+(5)	0.8480	0.8801	0.8640	0.7284	0.9400	0.9383
	(1)+(3)+(4)+(5)	0.8501	0.8822	0.8662	0.7327	0.9410	0.9373
	(2)+(3)+(4)+(5)	0.8501	0.8908	0.8704	0.7401	0.9371	0.9303
	All	0.8501	0.9015	**0.8758**	**0.7526**	**0.9487**	**0.9503**

Bold values indicate the best results.

### 3.2 Sequence composition analysis

**Figure 3:**
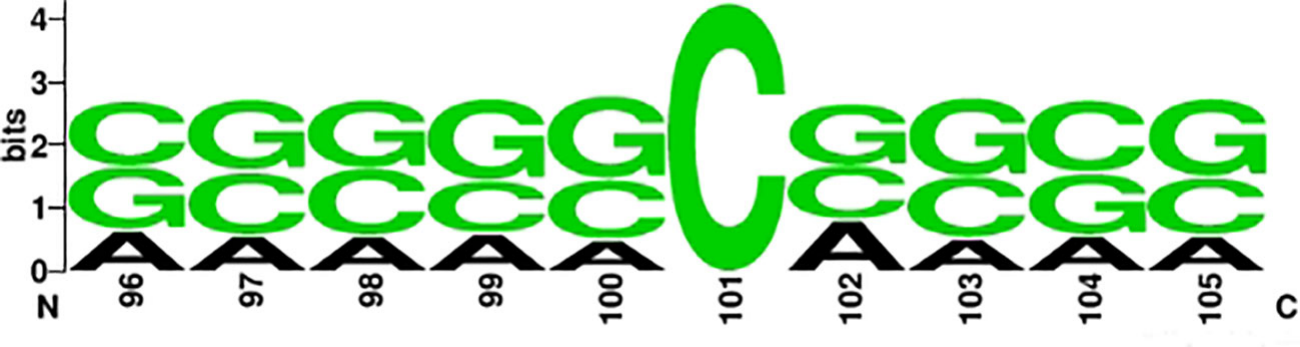
Nucleotide positive sample sequence diagram.

**Figure 4:**
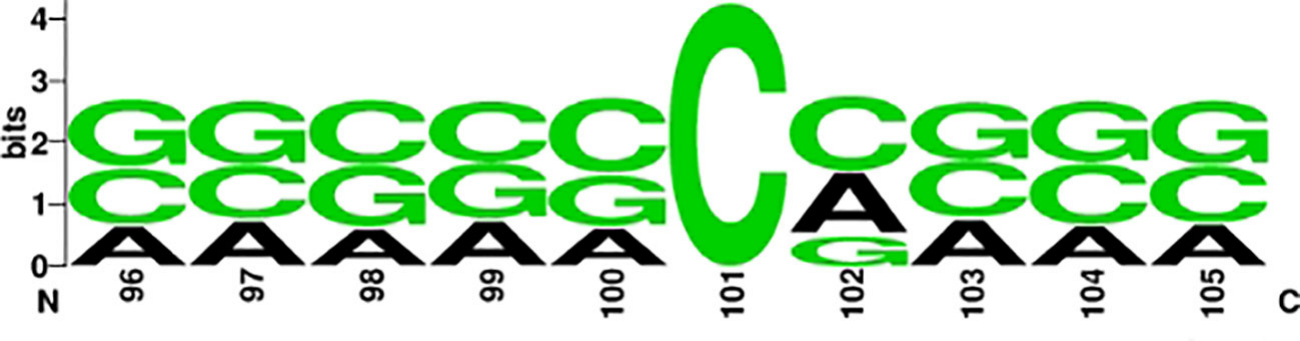
Nucleotide negative sample sequence diagram.

To investigate the distribution and preference of nucleotides of ac4C, we used the online tool Weblogo (45, 46) to mine the conserved motifs of ac4C sequences. **Figures 3** and **4** show the conserved motifs of the ac4C sequence and the distribution and preference of ac4C nucleotides. **Figure 4** is a highly enriched motif (CXX) in the ac4C-containing sequence, similar to the experimental results of Arango et al. (19). They utilize transcriptome-wide approaches to investigate ac4C localization and function in mRNA. They find that cytidine-containing mRNA codons are enriched in acetylated transcripts compared to other non-acetylated transcripts, which can enhance mRNA translation.

### 3.3 Stacking-ac4c model

The use of multiple heterogeneous models under appropriate integration strategies can achieve complementarity of models under training data compared to integration within a single model, thus significantly improving the reliability and efficiency of the model. In addition, machine learning models such as Logistic, KNN, SVM, RF, and MLP have been used in many bioinformatics fields and have made significant progress. Therefore, in the current study, a heterogeneous inheritance model stacking was used, 10 models were trained, and the simple averaging method was considered as the final result (**Table 6**).

**Table 6:** Performance of the ten models trained on the unbalanced dataset.

Cycle index	Validation results				Independent test results			
	Acc	Mcc	ROC	PRC	Acc	Mcc	ROC	PRC
1	0.9000	0.8008	0.9681	0.9702	0.8771	0.7548	0.9488	0.9624
2	0.8957	0.7910	0.9520	0.9558	0.8676	0.7153	0.9392	0.9511
3	0.9087	0.8157	0.9748	0.9708	0.8831	0.7767	0.9583	0.9688
4	0.9087	0.8175	0.9656	0.9622	0.8887	0.7797	0.9553	0.9584
5	0.9000	0.7994	0.9564	0.9605	0.8608	0.7486	0.9523	0.9542
6	0.8435	0.6869	0.9373	0.9374	0.8830	0.7464	0.9492	0.9396
7	0.8734	0.7474	0.9498	0.9353	0.8751	0.7402	0.9509	0.9323
8	0.8777	0.753	0.9483	0.9571	0.8862	0.7524	0.9472	0.9591
9	0.8777	0.751	0.9425	0.9288	0.8544	0.7517	0.9389	0.9243
10	0.8952	0.7866	0.9463	0.9556	0.8821	0.7603	0.9465	0.9501
Avg	0.8881	0.7749	0.9541	0.9534	0.8758	0.7526	0.9487	0.9503

**Figure 5:**
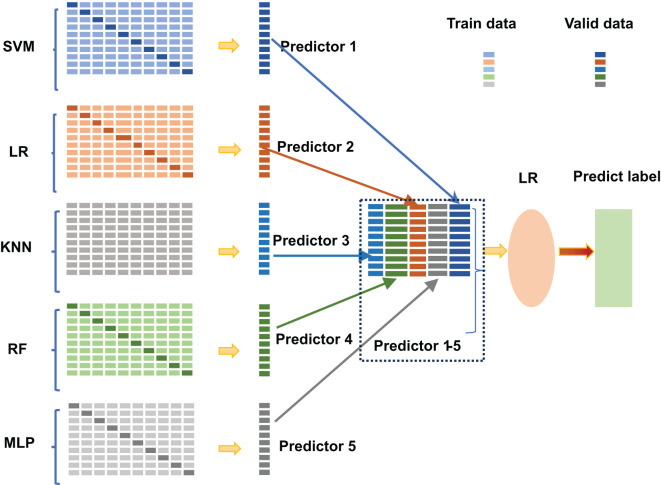
Framework diagram of Stacking ac4C stacked ensemble classifier.

The Stacking-ac4C model combined Kmer, PseEIIP, and PseKNC as the input of the model, as shown in **Figure 5**. The base model of the stacking model has SVM, Logistic, KNN, RF, and MLP composition, and the two-layer model meta-learner is LR after the training is completed; the model was validated. **Table 6** shows the Acc values of the model training results set and the model test results set’s Acc, Mcc, and ROC values. For the 10 unbalanced training datasets, the maximum Acc (0.9087) was obtained on Cycle 3 and Cycle 4, and the minimum Acc (0.8435) on Cycle 6. The maximum Acc (0.8862) was obtained on Cycle 8 and the minimum Acc (0.8544) on Cycle 9 for the independent test values. The relatively small variance of the training set and the independent test values indicate that the model is stable.

### 3.4 Comparison with published models

In this section, we will compare the proposed model with some existing ac4C site prediction models, namely PACES, XG-ac4C, and DeepAc4C. To validate the robustness and superiority of the proposed Stacking-ac4C, the three existing methods and our method were performed on the independent data set. As shown in **Table 7**, DeepAc4C shows a 7.53% improvement in ROC and a 15.61% improvement in Mcc, indicating that the Stacking-ac4C model has better imbalance data handling capability. Compared to XG-ac4C, the Stacking-ac4C model has an increase in ROC of 5.97% (**Table 7**), indicating that Stacking-ac4C has excellent stability and generalization ability. The low Sn and high Sp of PACES and XG-ac4C models may be due to the fact that the dataset used extracted specific motif sequences and ignored positive samples that did not match the feature. In addition, the reason for the lower Acc and Sp of DeepAc4C compared to XG-ac4C may be that the DeepAc4C model was trained with balanced data, but the test data was 1:10 unbalanced data.

**Table 7:** Results of independent tests of published models on unbalanced data sets.

Tools	Sn	Sp	Acc	Mcc	ROC	PRC
PACES	0.1513	0.8920	0.8835	0.2763	0.8741	0.4852
XG-ac4C	0.5824	**0.9439**	**0.9045**	0.4918	0.8890	0.5815
DeepAc4C	0.8222	0.7734	0.7979	0.5965	0.8734	0.8535
Stacking-ac4C	**0.8501**	0.9015	0.8758	**0.7526**	**0.9487**	**0.9503**

Bold values indicate the best results.

### 3.5 Comparison of results in the second data

**Figure 6:**
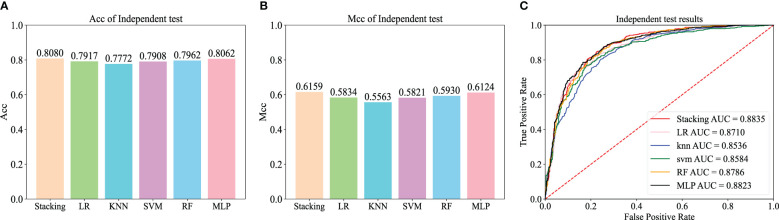
Independent test results on the balanced dataset of the basic machine learning model.

To validate the superior generalization ability of our tissue-Specific model compared to existing tools, a dataset was constructed for testing iRNA-ac4C. The ratio of positive to negative samples in this dataset is 1:1. These datasets are available from http://lin-group.cn/server/iRNA-ac4C/ (10). We performed independent tests on the four existing methods along with Stacking-ac4c, and the results of the final independent tests are listed in **Table 8**. The corresponding ROC curves are shown in **Figure 6C**. All models showed relatively high results in Sp but relatively low results in Sn, especially the first three models, which may be due to training on a highly unbalanced dataset, where two models learned more information from negative samples than from positive samples. In addition, compared with iRNA-ac4C, the Sn of Stacking-ac4C increased by 4.1%, which indicates the high sensitivity of the model; the Acc increased by nearly 1%, which indicates a more Accurate model; and the ROC increased by 0.49%, which indicates the superior stability and generalization ability of the Stacking-ac4C model. Meanwhile, the Stacking ac4C model is used in the dataset http://www.rnanut.net/paces/ and datasets http://lin-group.cn/server/iRNA-ac4C/. The above results are superior to other models, indicating that this model has good generalization ability.

**Table 8:** Results of independent tests of published models on balanced data sets.

Tools	Sn	Sp	Acc	Mcc	ROC
PACES	0.0598	**1.0000**	0.5299	0.1760	\
XG-ac4C	0.3587	0.8243	0.5915	0.2070	\
DeepAc4C	0.1007	0.9710	0.5362	0.1470	0.8030
iRNA-ac4C	0.7670	0.8291	0.7981	0.5970	0.8800
Stacking-ac4C	**0.8080**	0.8080	**0.8080**	**0.6159**	**0.8835**

Bold values indicate the best results.

To further evaluate the effectiveness of the Stacking architecture, we compared Stacking with five popular traditional machine learning algorithms, including logistic regression (LR), k-nearest neighbor (KNN), support vector machine (SVM), random forest (RF), and multilayer perceptron (MLP) algorithms. For a fair comparison, the models were trained using the dataset constructed from the iRNA-ac4C article and evaluated using an independent test dataset. **Figures 6A, B** show the Acc and Mcc of five popular traditional machine learning algorithms, and **Figure 6C** shows the ROC of five popular traditional machine learning algorithms. This shows that Stacking-ac4C achieves the best scores on Acc, Mcc, and ROC, indicating that compared to traditional classifiers, the proposed model outperforms the ac4C recognition of traditional classifiers.

## 4 Summary

Four models were built to identify the ac4C locus in human mRNA. However, there is still room to improve the performance of these predictors. In this study, a new predictor, Stacking-ac4C, is developed, which utilizes three coding methods (i.e., Kmer, PseKNC, and PseEIIP) and uses the Stacking-based algorithm in the classification method to identify ac4C sites. In addition, the results tested in an independent test set of PACES article data showed that Stacking-ac4C outperformed other existing tools. Similarly, the testing results in an independent test set of iRNA-ac4C article data showed that Stacking-ac4C also outperformed other existing tools. The benchmark test dataset and source code can be downloaded from https://github.com/louliliang/ST-ac4C.git. However, the proposed model still has some shortcomings. First, Stacking integrated learning, despite improving the sensitivity of the model in predicting real ac4C loci, lacks the application of a deep learning model on ac4C loci compared to DeepAc4C. Secondly, although the model achieved good results on both PACES and iRNA-ac4C data, the accuracy of the model in the PACES dataset appeared to be insufficient compared to the XG-ac4C model. In addition, a comparison of **Tables 7** and 8 shows that the model is suitable for dealing with unbalanced datasets specific to motifs, and the enhancement effect on balanced baseline datasets is not great. In future work, we will further experiment with other approaches to enable our model to outperform existing predictors. In conclusion, Stacking-ac4C is an effective tool for identifying ac4C sites in mRNA and contributes to our functional understanding of ac4C in RNA.

## Data availability statement

Information for existing publicly accessible datasets is contained within the article.

## Ethics statement

The manuscript presents research on animals that do not require ethical approval for their study.

## Author contributions

L-LL: Writing – original draft, Data curation. W-RQ: Conceptualization, Project administration, Supervision, Writing – review & editing. ZL: Software, Validation, Writing – original draft. Z-CX: Methodology, Validation, Writing – review & editing. XX: Funding acquisition, Writing – review & editing. S-FH: Supervision, Writing – review & editing.
