# Supplementary material for: Stacking-ac4C: an ensemble model using mixed features for identifying n4-acetylcytidine in mRNA
Source: Front Immunol. 2023 Nov 29;14:1267755. doi: 10.3389/fimmu.2023.1267755 (PMC10716444; doi:10.3389/fimmu.2023.1267755)
Supplement: Supplementary file 1 [file DataSheet_1.pdf]

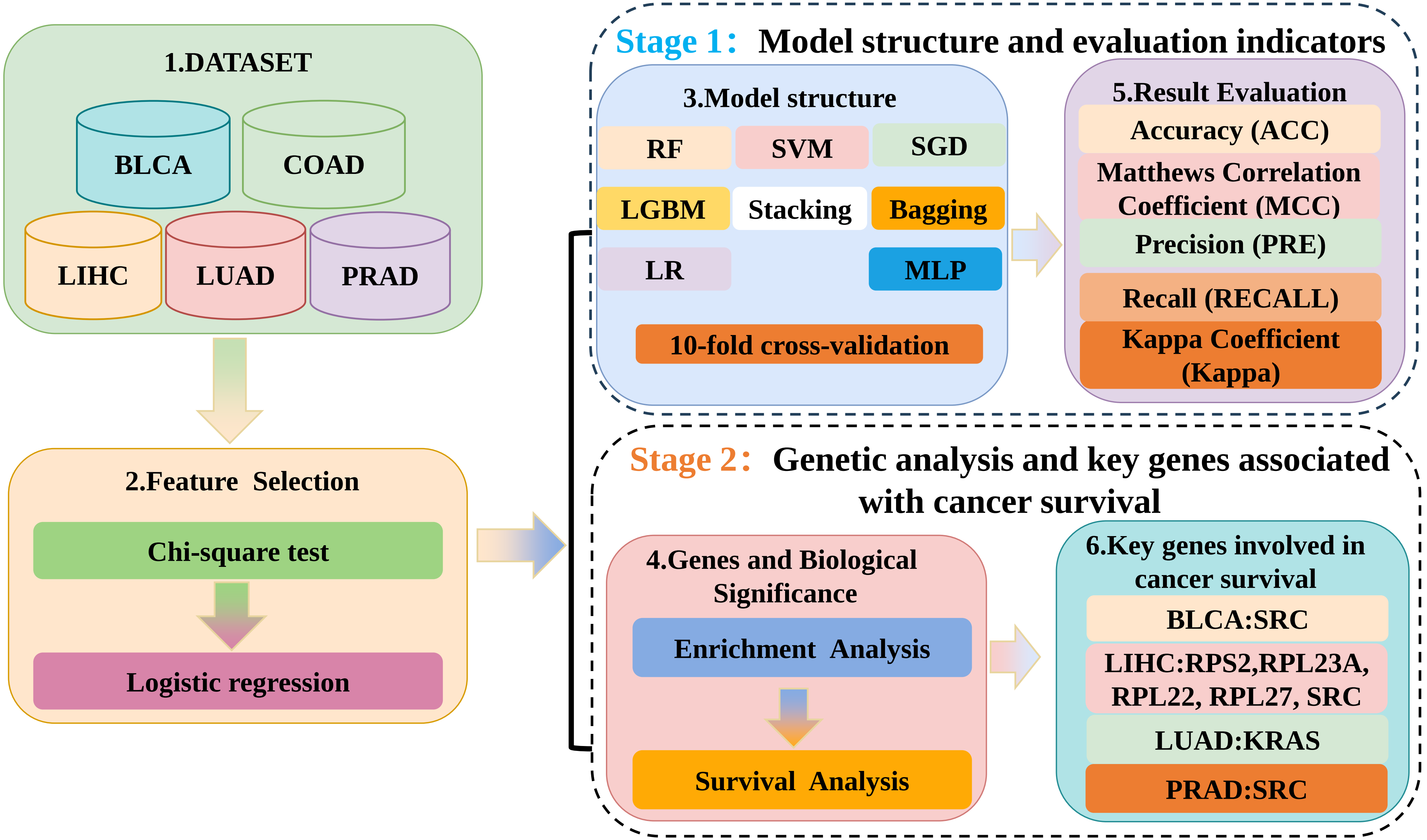

Figure 1. Overall workflow diagram

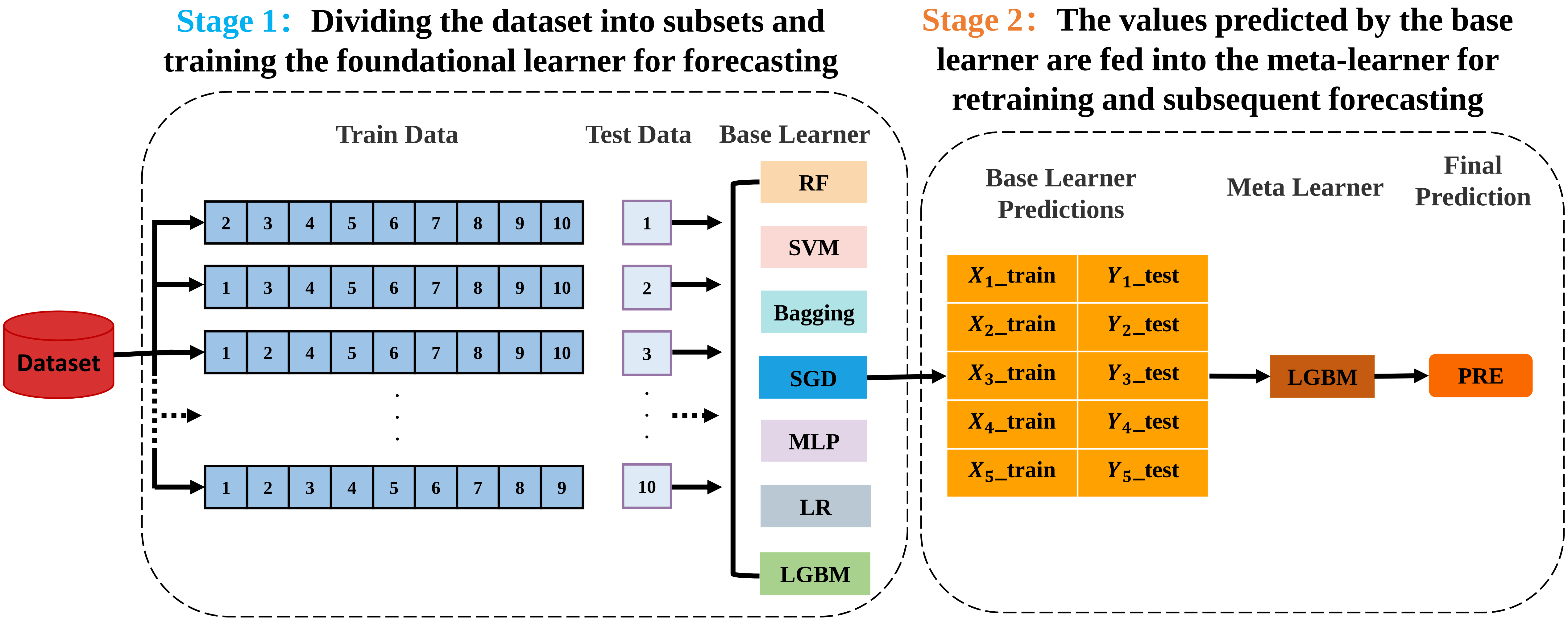

Figure 2. Stacking ensemble Modeling Framework

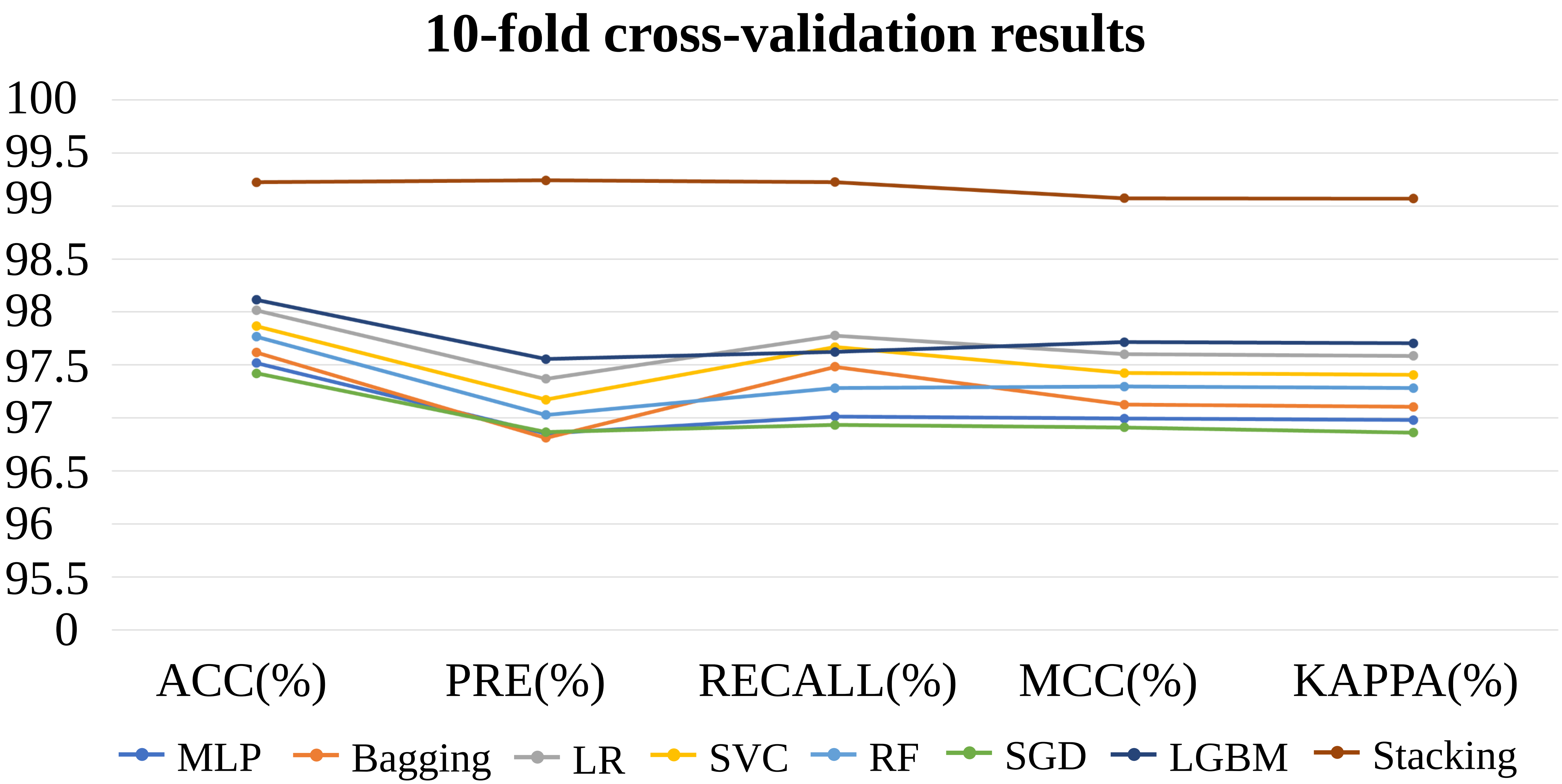

Figure 3(a). 10-fold cross-validation results and Average results of 10 independent tests line graph

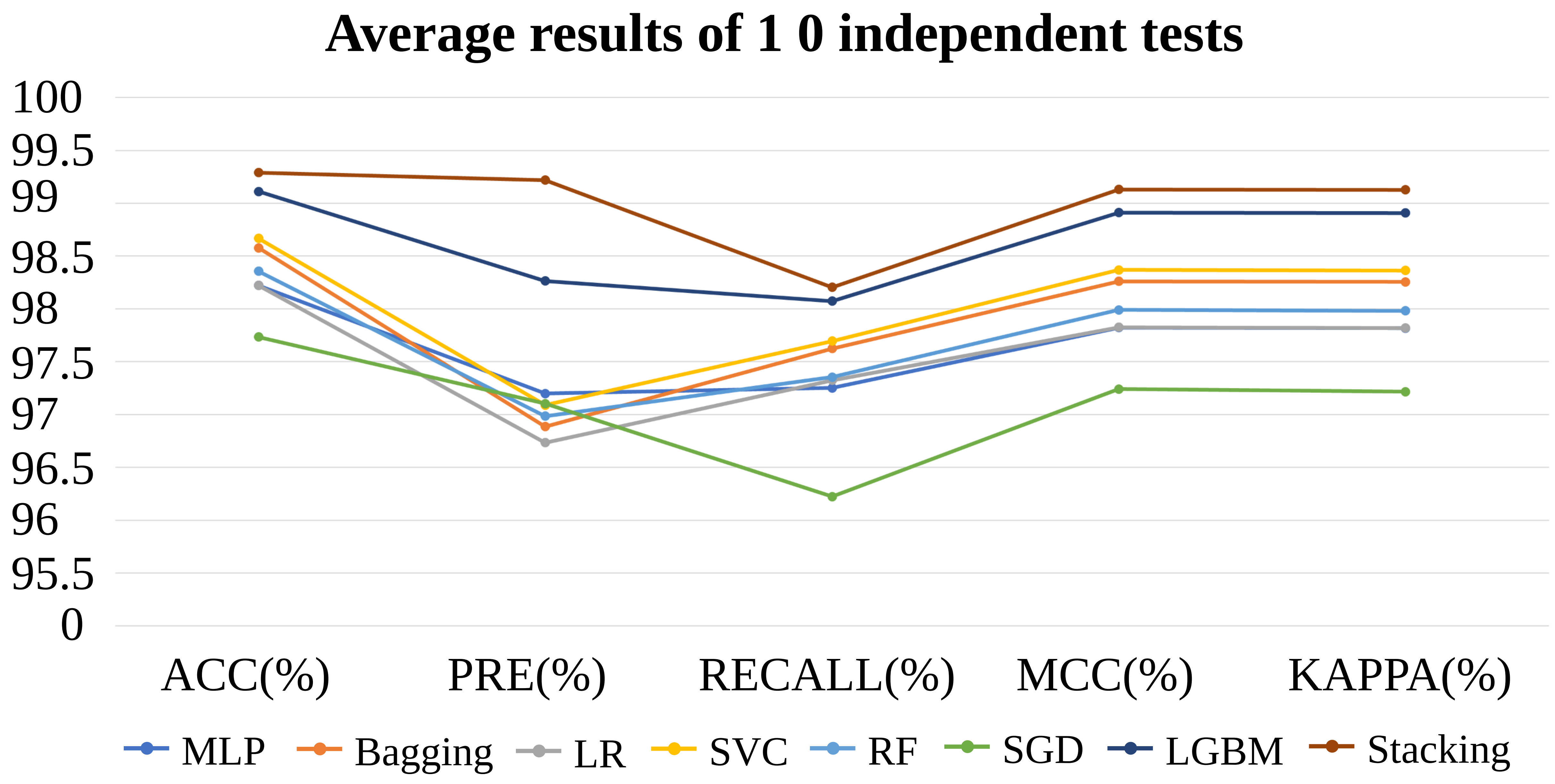

Figure 3(b). 10-fold cross-validation results and Average results of 10 independent tests line graph

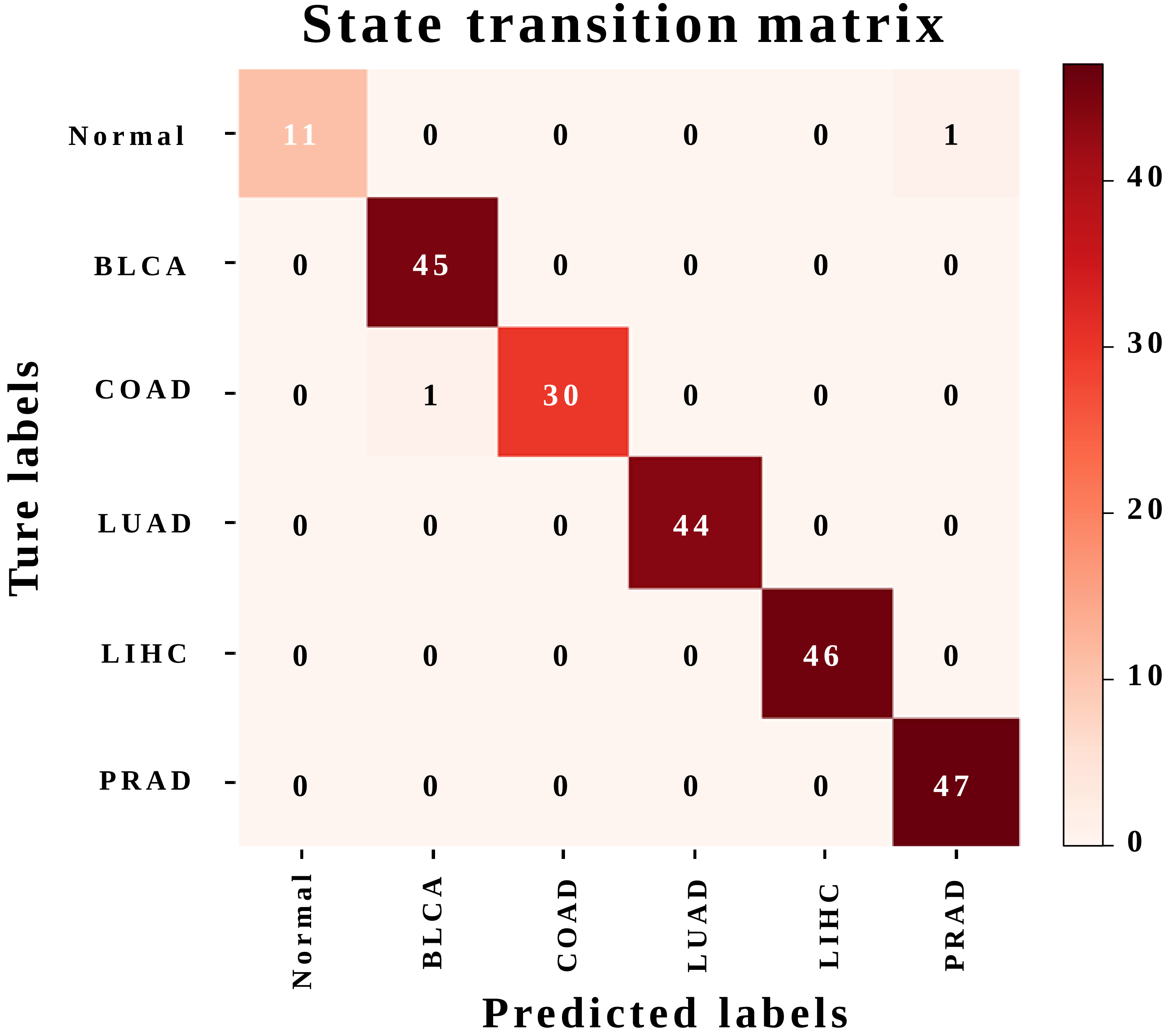

Figure 4. Confusion matrix for independent testing of multi-class predictors

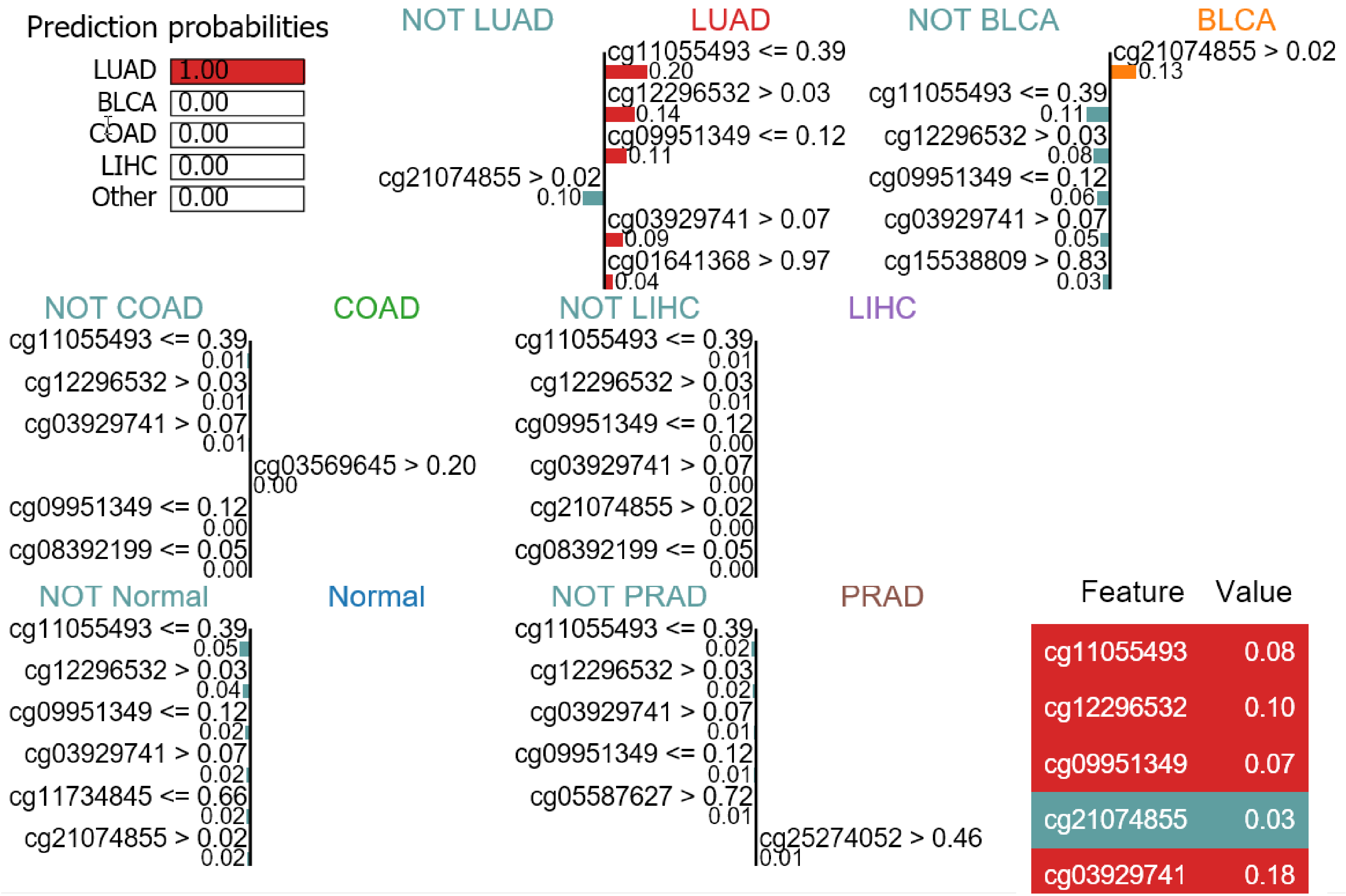

Figure 5. LIME results of 6 major biomarkers illustrated using Stacking ensemble learning classifier for normal tissue (Normal), bladder urothelial carcinoma (BLCA), colon adenocarcinoma (COAD), liver hepatocellular carcinoma (LIAD), lung adenocarcinoma (LUAD), and prostate adenocarcinoma (PRAD)； LIME：Local Interpretable Model-Agnostic Explanations

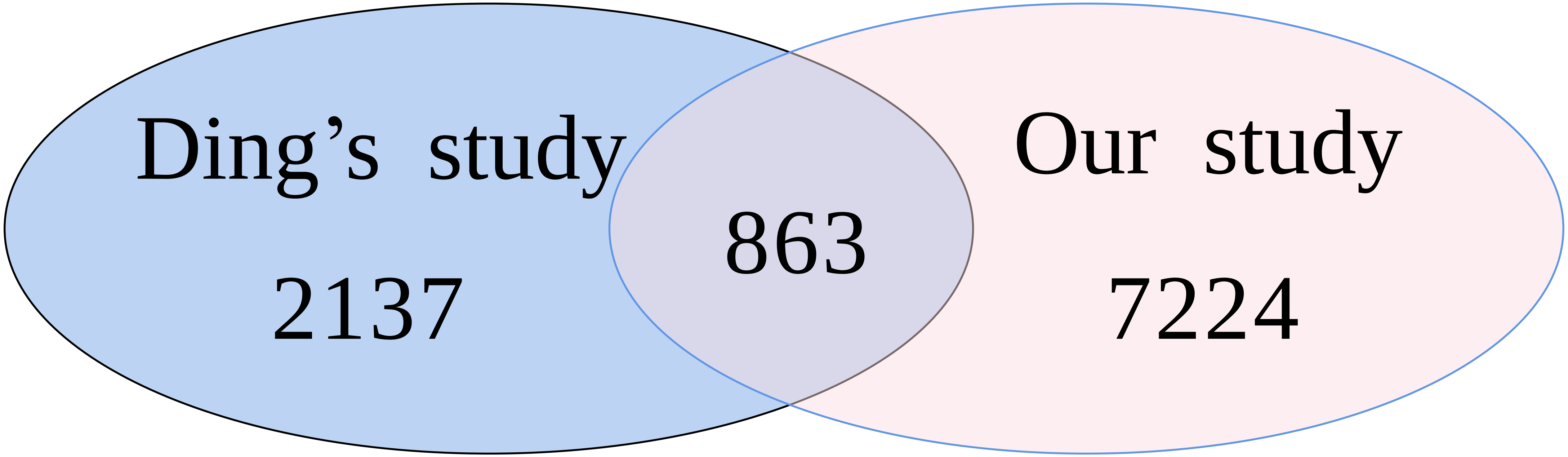

Figure 6. VENN maps of overlapping genes between the two studies Venn

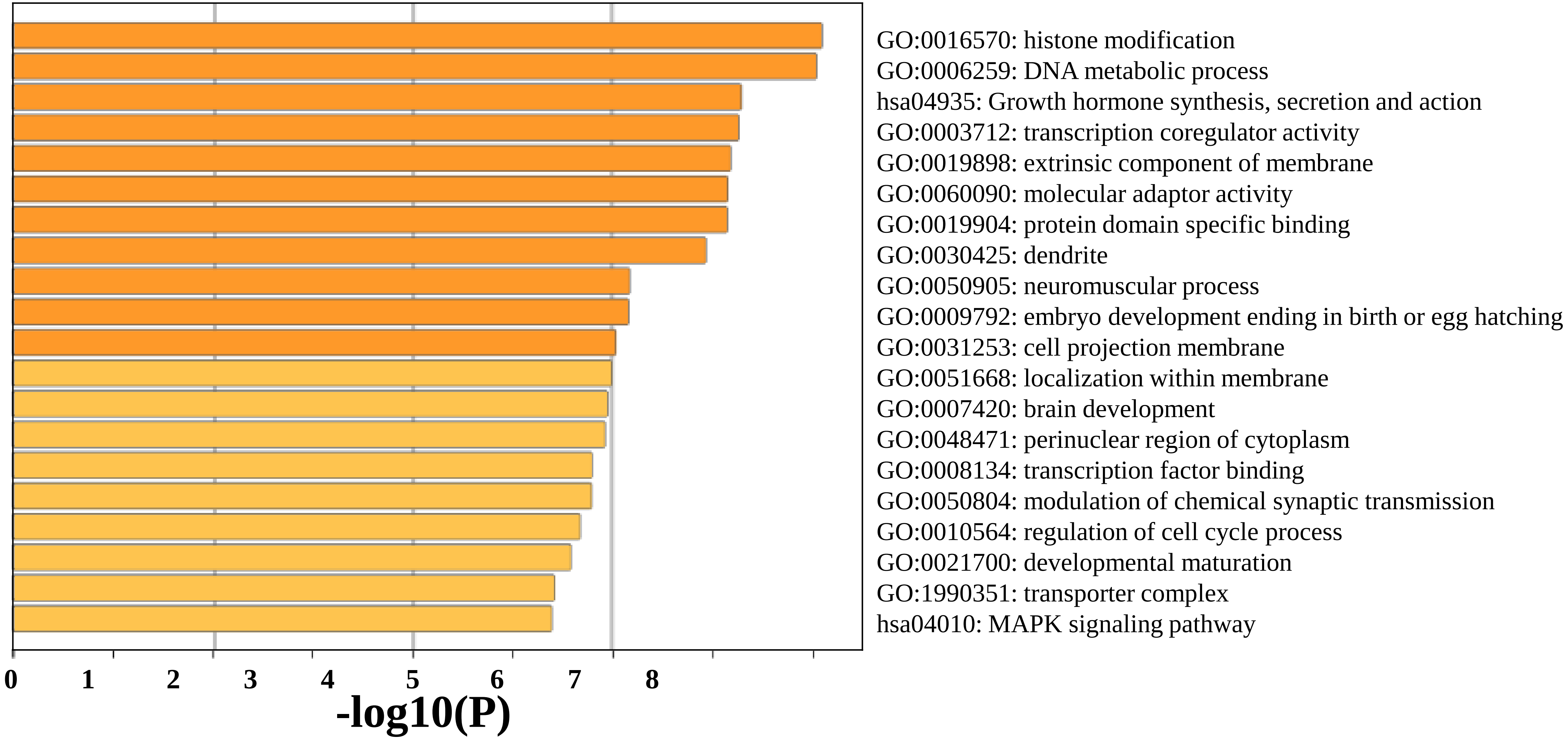

Figure 7. Heatmap of enriched terms for overlapping genes. The intensity of the color indicates the level of enrichment, with darker colors indicating higher levels of enrichment. On the right side, there is a wealth of information on terms from the Gene Ontology (GO) and KEGG Pathway that can be used to clarify the meaning and function of each enrichment term

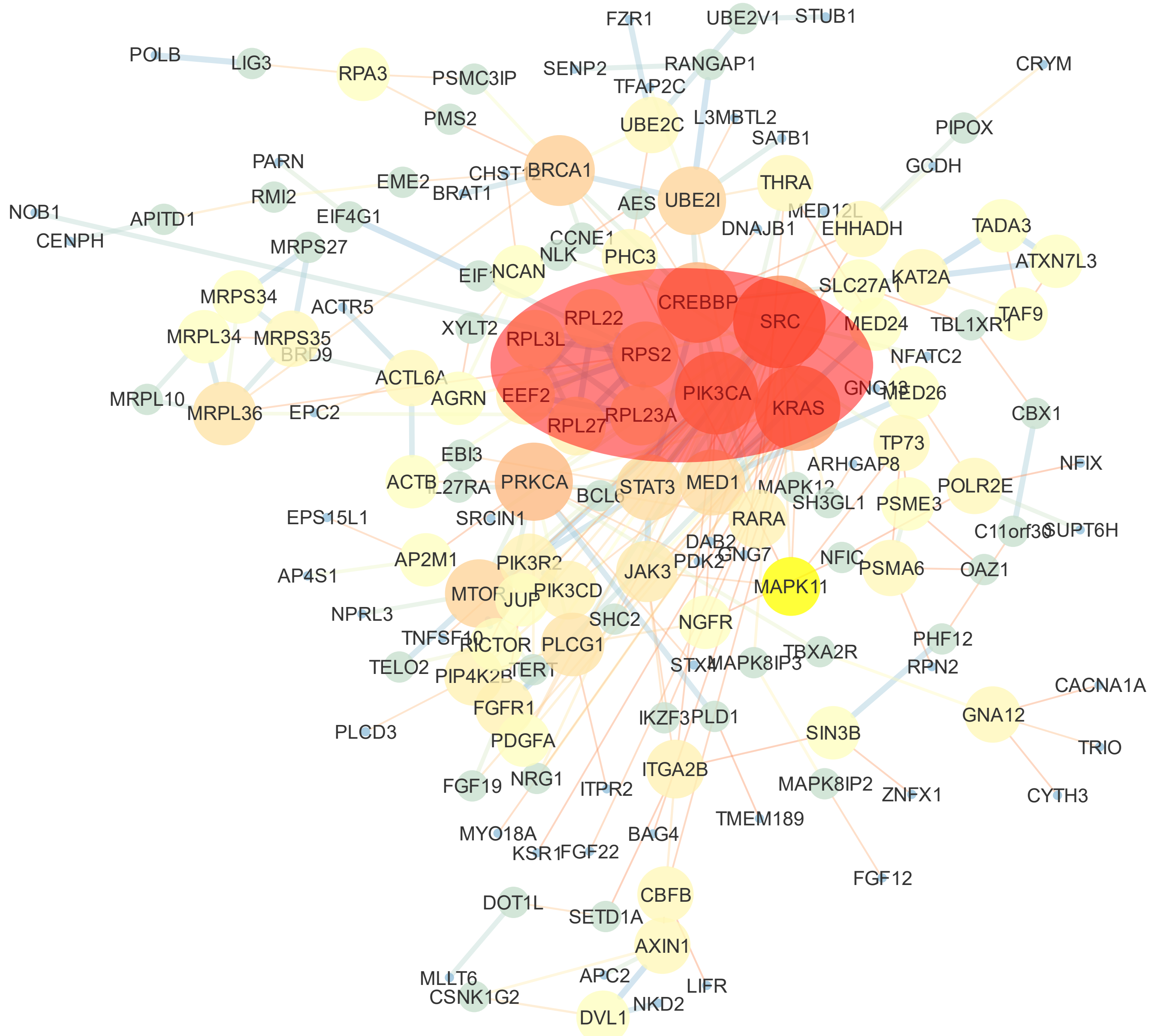

Figure 8. Protein-protein interaction networks generated by overlapping gene lists. The genes marked in red are the top 10 key genes scored by the MCC method（RPS2, RPL23A, RPL3L, RPL22, RPL27, EEF2, PIK3CA, SRC, KRAS, and CREBBP）

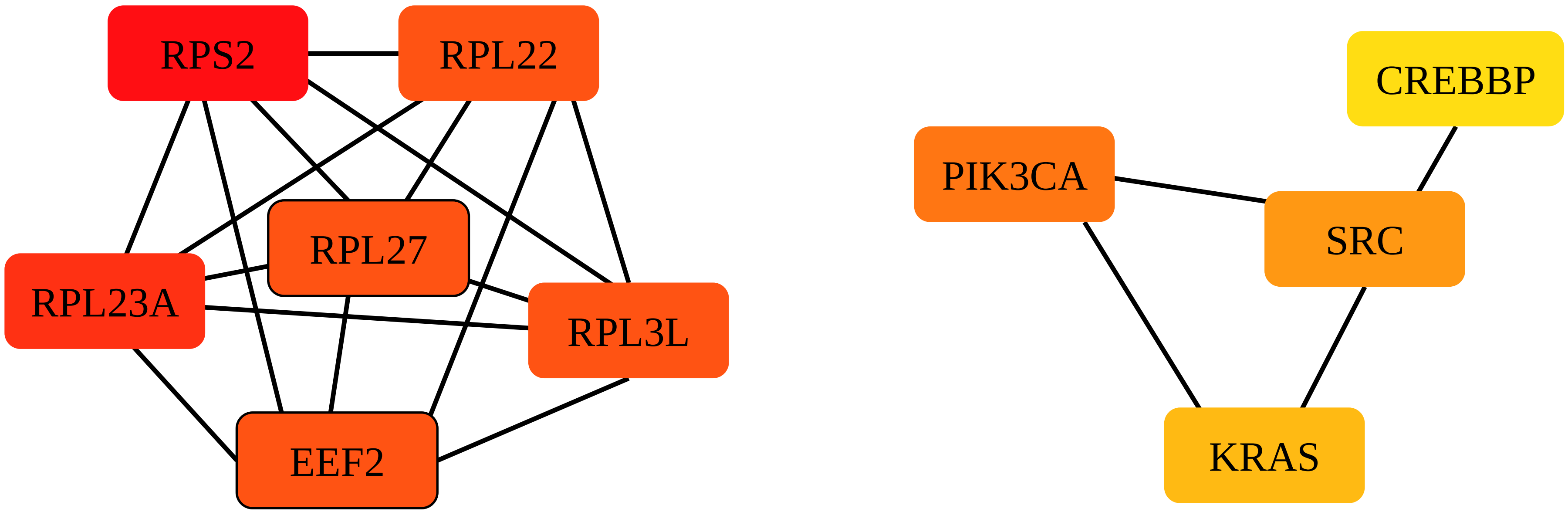

Figure 9. Key genes for scoring top 10

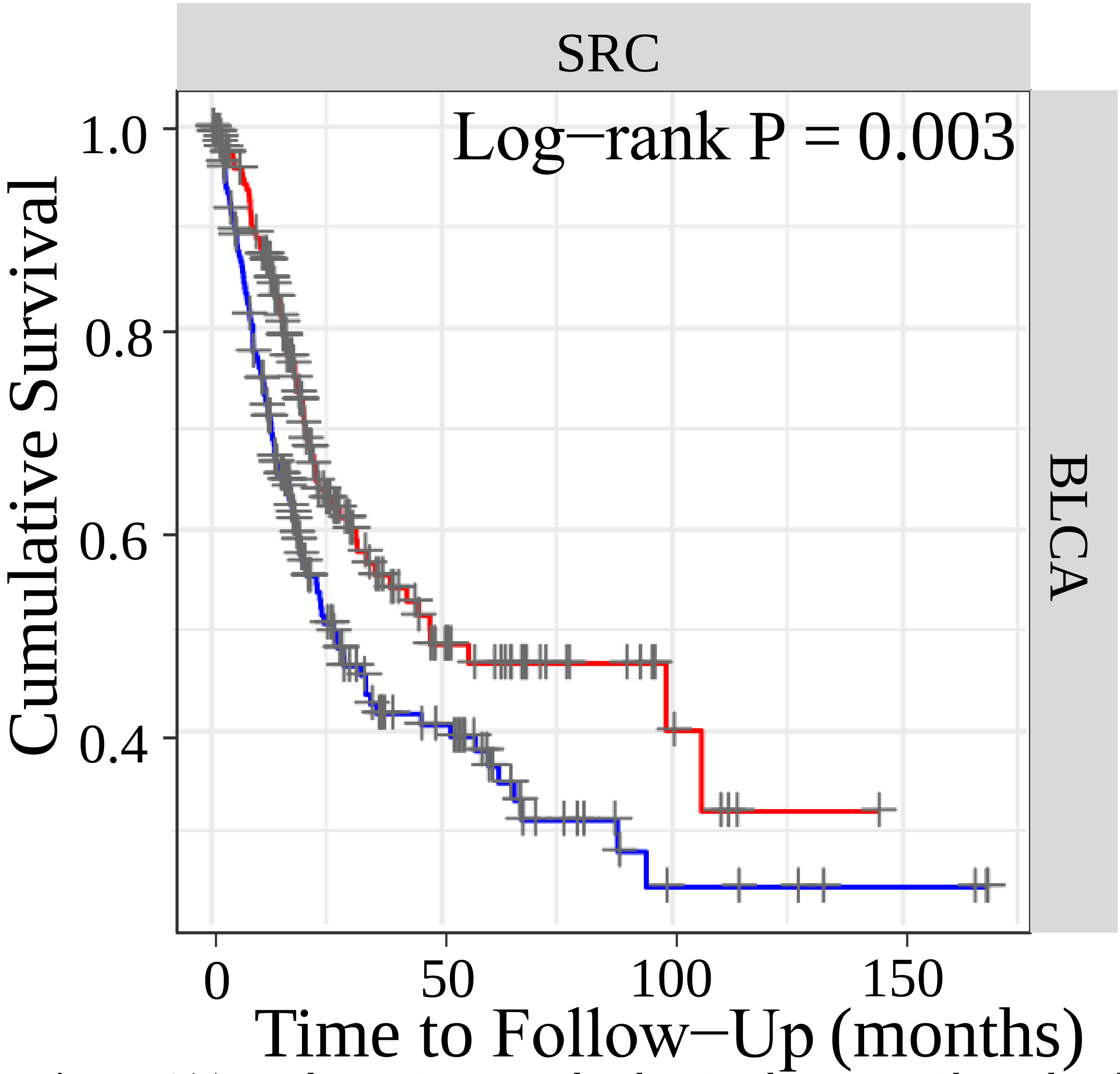

Figure 10(a). Kaplan-Meier curve plot showing the genes with p-values less than 0.05

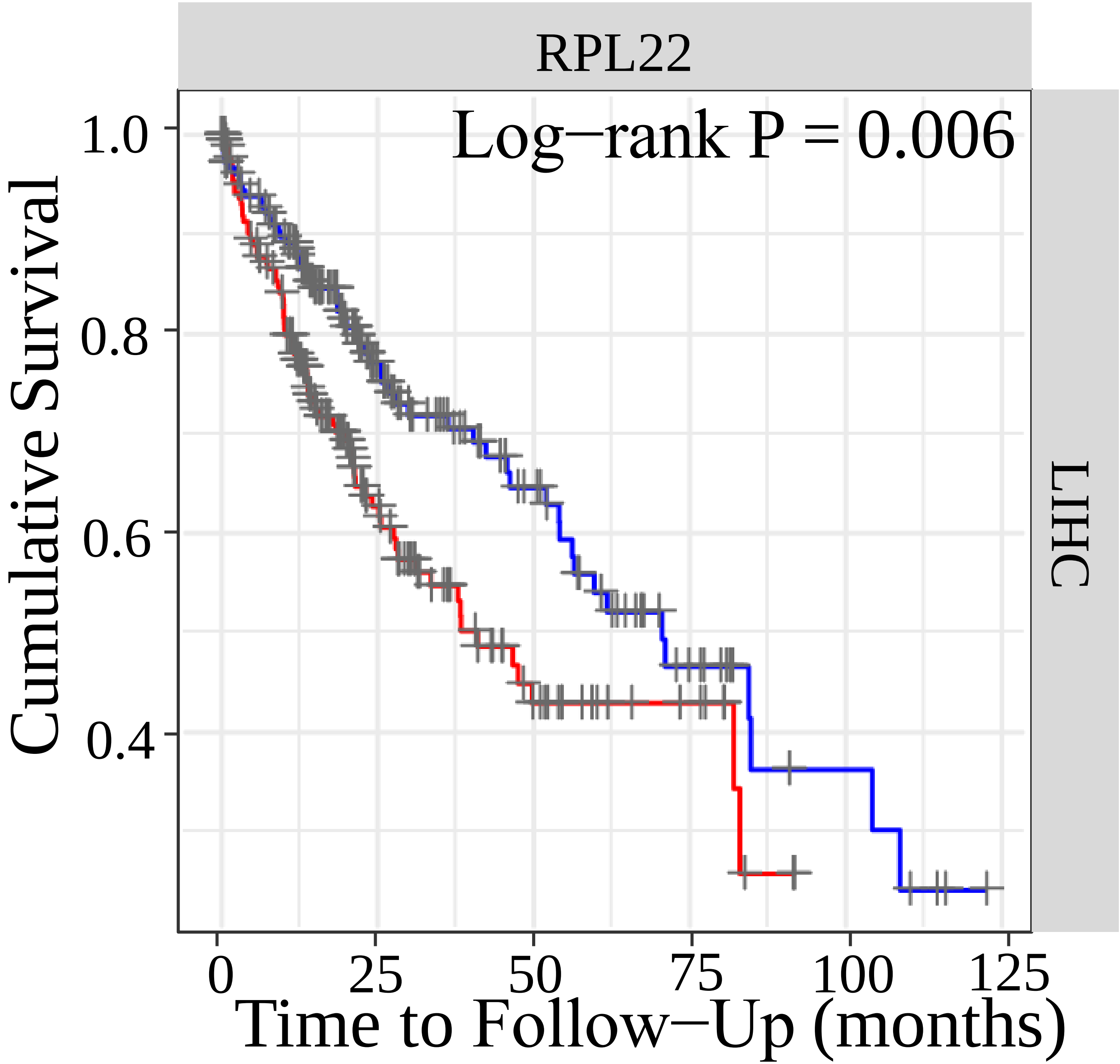

Figure 10(b). Kaplan-Meier curve plot showing the genes with p-values less than 0.05

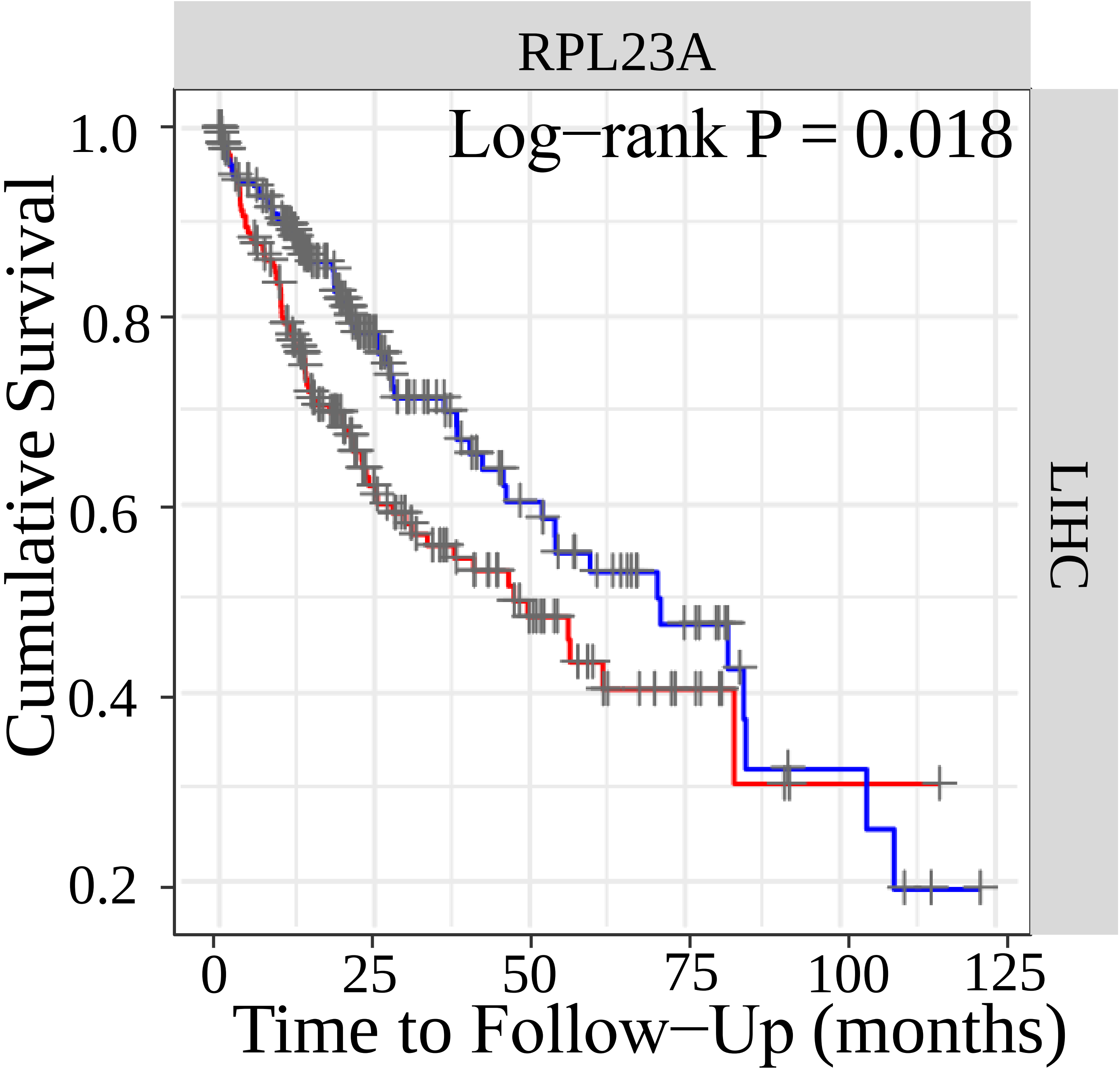

Figure 10(c). Kaplan-Meier curve plot showing the genes with p-values less than 0.05

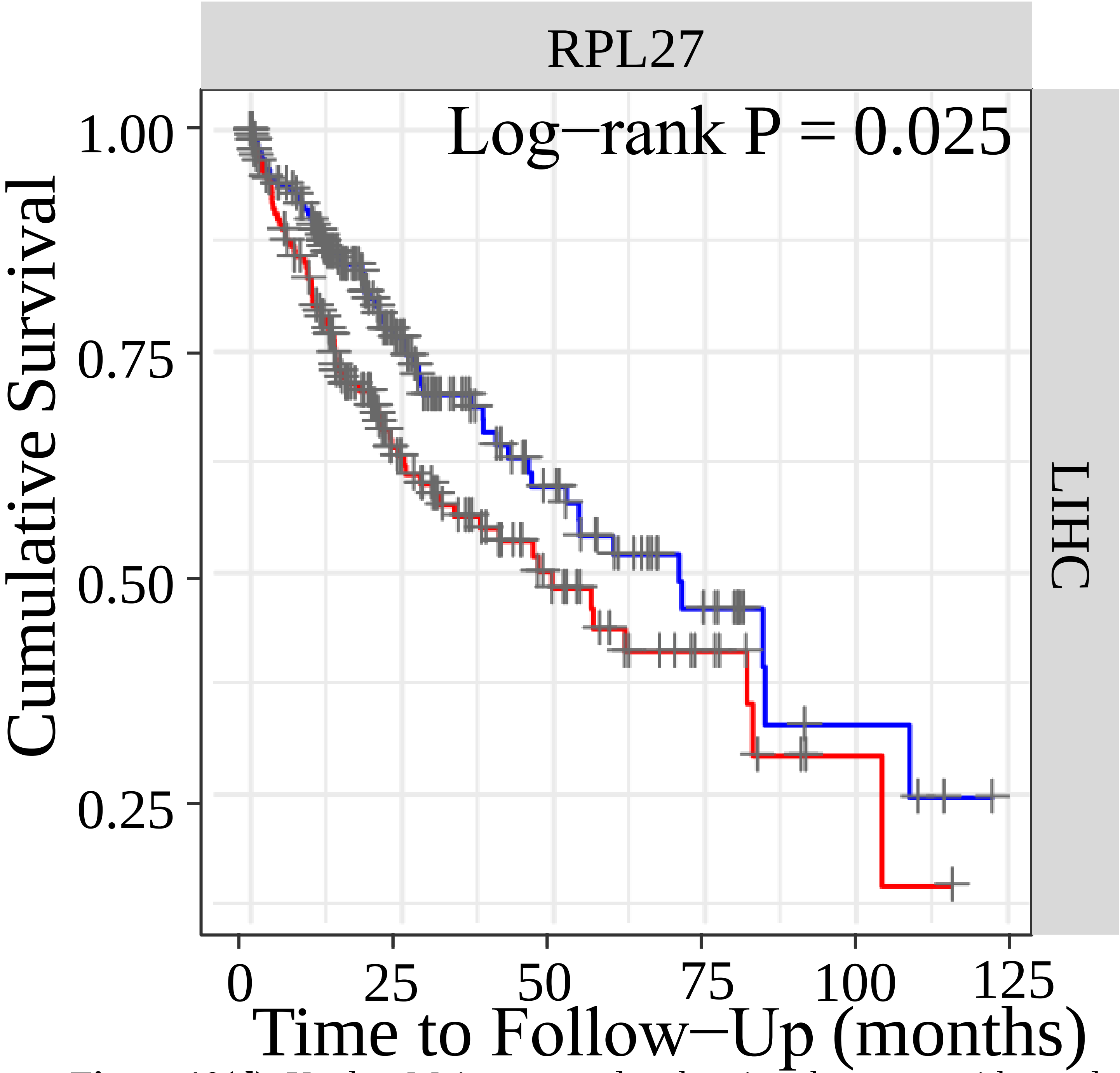

Figure 10(d). Kaplan-Meier curve plot showing the genes with p-values less than 0.05

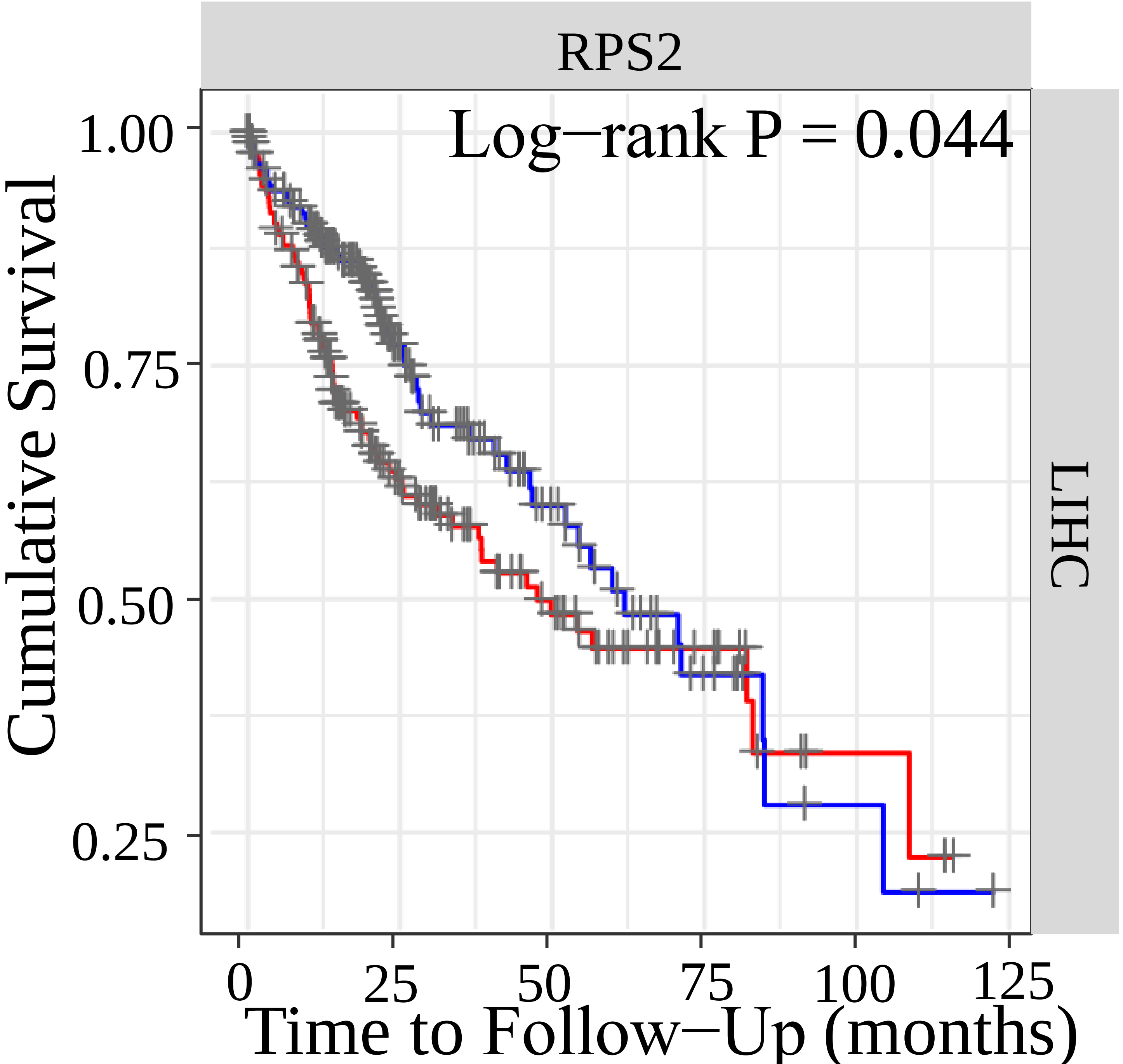

Figure 10(e). Kaplan-Meier curve plot showing the genes with p-values less than 0.05

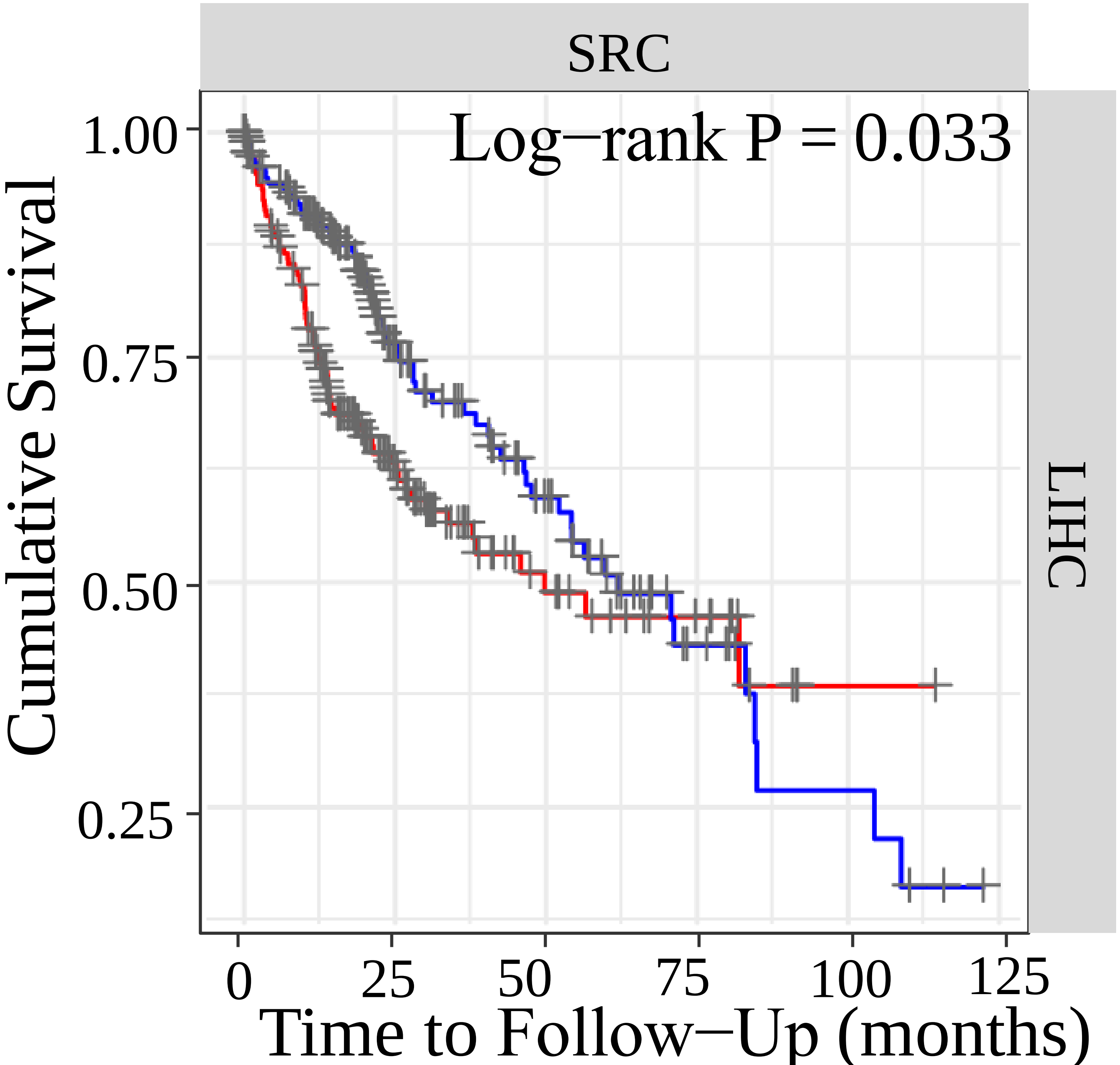

Figure 10(f). Kaplan-Meier curve plot showing the genes with p-values less than 0.05

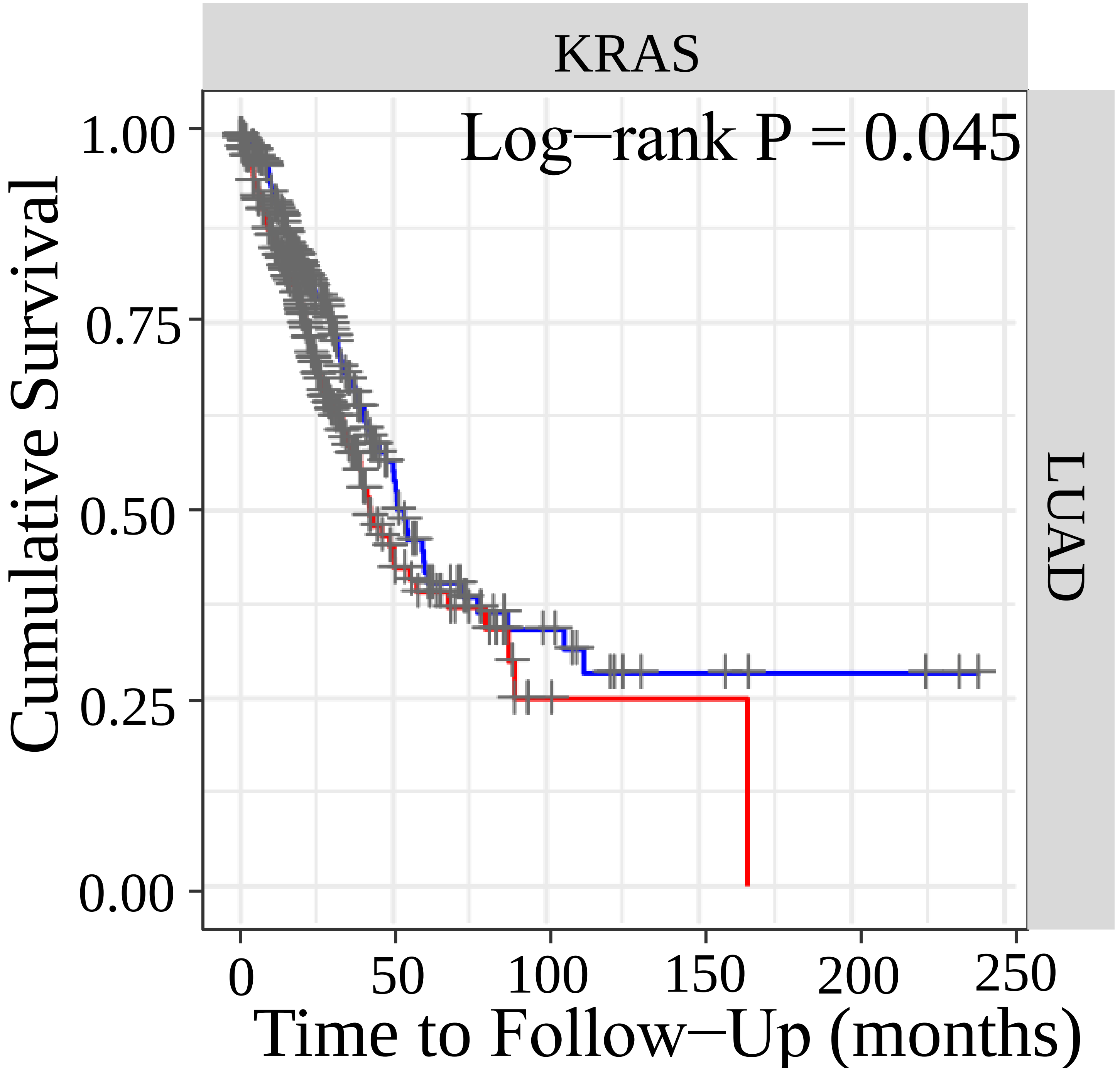

Figure 10(g). Kaplan-Meier curve plot showing the genes with p-values less than 0.05

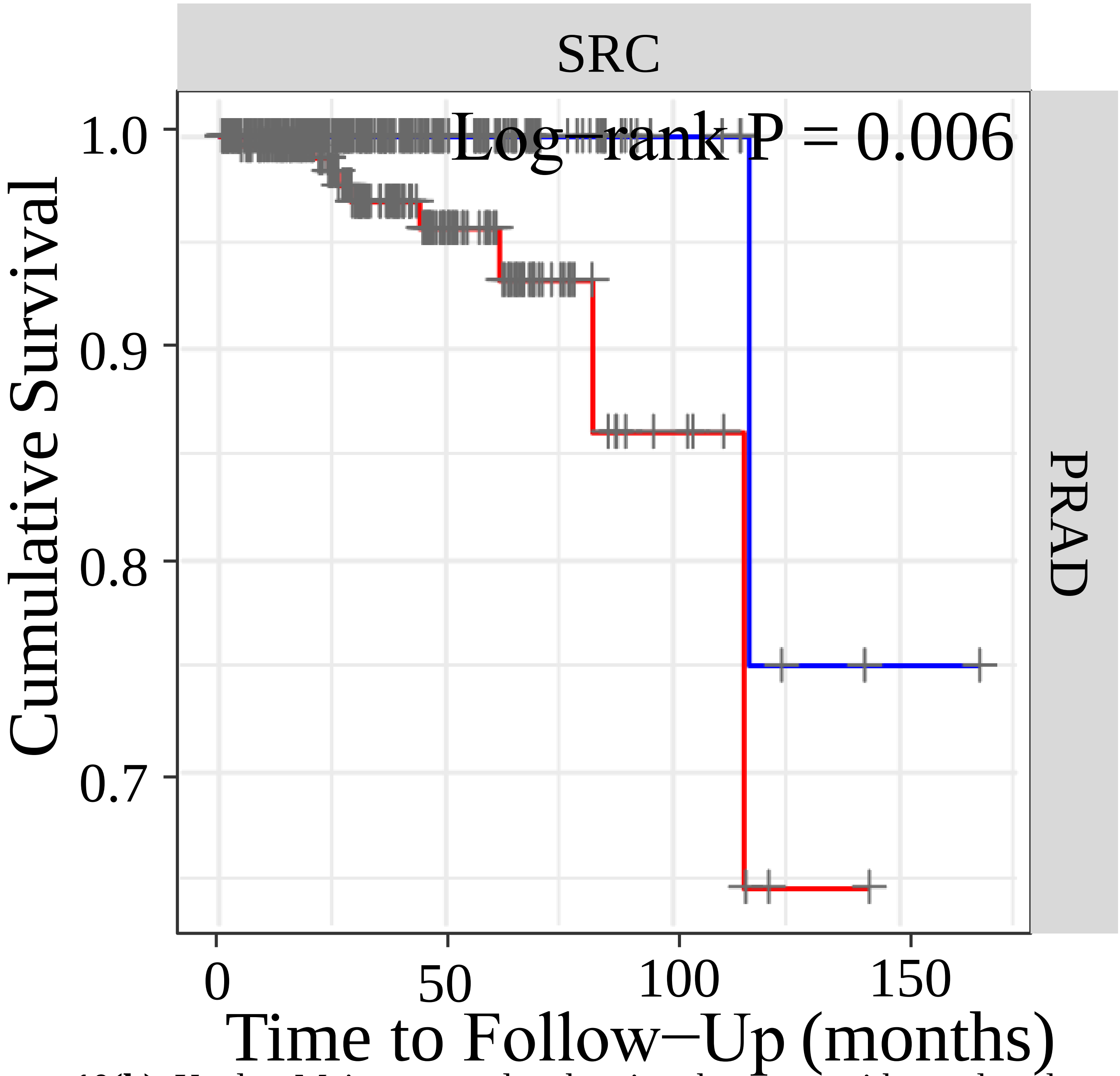

Figure 10(h). Kaplan-Meier curve plot showing the genes with p-values less than 0.05

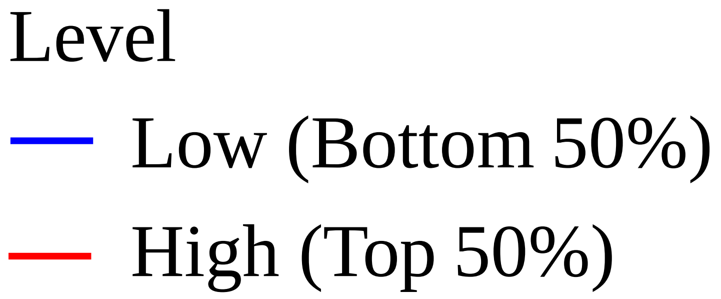
Level
Low (Bottom 50%)
High (Top 50%)
